# The Case of a 37‐Year‐Old Woman Presenting With Subacute Weakness and Paresthesias

**DOI:** 10.1002/acn3.70289

**Published:** 2025-12-17

**Authors:** Peter Pacut, Joy Cannon, Neel Dixit, Qihua Fan

**Affiliations:** ^1^ PeaceHealth Neurology Virginia Commonwealth University Richmond Virginia USA

**Keywords:** acute intermittent porphyria, Guillain Barre mimics motor neuropathy, subacute weakness, wrist drop

## Abstract

Acute intermittent porphyria (AIP) is a rare metabolic disorder that may present with subacute neuropathy and systemic symptoms, often leading to diagnostic delay. We report a 37‐year‐old woman with eight weeks of progressive bilateral upper extremity weakness and paresthesias, followed by lower extremity involvement and falls, in the setting of chronic abdominal pain, presyncope, weight loss, and neuropsychiatric symptoms. Examination revealed profound proximal arm weakness, sensory deficits, bulbar involvement, and autonomic features. MRI of the brain and spine and cerebrospinal fluid analysis were normal. Electrodiagnostic studies demonstrated a severe diffuse motor neurogenic process. Markedly elevated urinary porphobilinogen and aminolevulinic acid levels confirmed the diagnosis of acute intermittent porphyric neuropathy, supported by identification of a variant of uncertain significance in the HMBS gene. This case underscores the importance of considering AIP in patients with subacute motor‐predominant neuropathy accompanied by abdominal pain and autonomic dysfunction, as early diagnosis enables timely treatment and improved outcomes.

## 1 Summary of Case

A 37‐year‐old woman presented with 8 weeks of progressive bilateral upper extremity weakness and paresthesias, in the context of 1 year of intermittent abdominal pain and frequent presyncope. Additional symptoms included hoarseness, dysphagia, a 30‐lb weight loss over 2 months, and anxiety.

– Neurologic symptoms: Initial upper extremity weakness progressed to involve the lower extremities, resulting in falls. Sensory deficits involved the trunk and extremities.
– Systemic symptoms: Poor appetite, intermittent fevers, constipation, lightheadedness, and fatigue.
– Exam findings: Anxious appearance, tachycardia, hoarse voice, weak cough. Motor exam revealed profound proximal arm weakness (0/5), mild distal hand and lower limb weakness (1–4/5). Reflexes were absent in the upper limbs and preserved in the lower limbs. Sensory loss to light touch was present.

### 1.1 Diagnostic Workup

– Serum Studies: Mild hyponatremia, elevated AST/ALT, negative for inflammatory, infectious, or autoimmune markers.
– MRI brain and spine and spinal fluid analysis were normal.
– EMG/NCS showed a severe diffuse motor neurogenic process.
– Porphyrin testing showed markedly elevated urinary porphobilinogen (PBG), ALA, and ALA/Cr ratio.
– Genetic Testing: Variant of uncertain significance in *HMBS*, a gene associated with acute intermittent porphyria.

## 2 Diagnosis

Acute intermittent porphyric neuropathy.

## 3 Take‐Home Points

– AIP should be suspected in cases of subacute weakness with autonomic dysfunction, abdominal pain, and neuropsychiatric symptoms [1].
– AIP neuropathy preferentially affects the radial nerve (wrist drop) and common peroneal nerve (foot drop) [2].
– Treatment includes glucose, hemin, and avoidance of precipitating factors [3].

## Author Contributions

Peter Pacut and Qihua Fan wrote the manuscript. Neel Dixit and Joy Cannon reviewed and edited the manuscript. All authors reviewed and approved the final version.

## Funding

The authors have nothing to report.

## Conflicts of Interest

The authors declare no conflicts of interest.

## Data Availability

The authors have nothing to report.
